# Decision-making, cognitive functions, impulsivity, and media multitasking expectancies in high versus low media multitaskers

**DOI:** 10.1007/s10339-021-01029-2

**Published:** 2021-05-28

**Authors:** Silke M. Müller, Johannes Schiebener, Matthias Brand, Magnus Liebherr

**Affiliations:** 1 Department of General Psychology: Cognition and Center for Behavioral Addiction Research (CeBAR), University of Duisburg-Essen, Forsthausweg 2, 47057 Duisburg, Germany; 2 Erwin L. Hahn Institute for Magnetic Resonance Tomography, Essen, Germany

**Keywords:** Media multitasking, Decision-making, Iowa Gambling Task, Game of Dice Task, Impulsivity, Expectancies, Internet addiction

## Abstract

In several studies, individuals who reported to frequently multitask with different media displayed reduced cognitive performance, for example in fluid intelligence and executive functioning. These cognitive functions are relevant for making advantageous decisions under both objective risk (requiring reflection and strategical planning) and ambiguous risk (requiring learning from feedback). Thus, compared to low media multitaskers (LMMs), high media multitaskers (HMMs) may perform worse in both types of decision situations. The current study investigated HMMs and LMMs in a laboratory setting with the Game of Dice Task (GDT; objective risk), the Iowa Gambling Task (IGT; ambiguous risk), various tests quantifying cognitive functions (logical reasoning, working memory, information processing, general executive functions), and self-report measures of impulsivity, media multitasking expectancies, and problematic Internet use. From 182 participants, 25 HMMs and 19 LMMs were identified using the Media Multitasking Index. Results show that HMMs compared to LMMs performed weaker on the IGT but not on the GDT. Furthermore, HMMs had slightly decreased performance in tests of logical reasoning and working memory capacity. HMMs tended to increased information processing speed but this difference was not significant. Furthermore, HMMs have more positive expectancies regarding media multitasking and reported higher tendencies toward problematic Internet use. HMMs and LMMs did not differ significantly with respect to impulsivity and executive functions. The results give a first hint that HMMs may have difficulties in decision-making under ambiguous but not under objective risk. HMMs may be more prone to errors in tasks that require feedback processing. However, HMMs appear not to be impaired in aspects of long-term strategic decision-making.

## Introduction

We watch movies while chatting with friends, we write e-mails while calling a colleague, or we check our smartphone while having a conversation. Today, mobile devices, such as smartphones and tablets, enable individuals to use media in parallel to other activities—a phenomenon that is often described as media multitasking (Cain et al. 2016; Foehr et al. 2005; Ophir et al. 2009). Thereby, the activity performed in parallel to media use can include either other media (e.g., listening to the radio while reading the newspaper), the same medium (e.g., doing work at the computer while listening to music from the computer), or no medium (e.g., checking the smartphone while driving) (Lang and Chrzan 2015). These behaviors might have some advantages, for example, a (perceived) increase of efficiency, a quick retrieval of relevant information, a potential reduction of boredom, as well as an experience of fun and pleasure (Hwang et al. 2014). At the same time, one might also think of disadvantages, as multitasking can induce stress (Wetherell and Carter 2014) or reduce speed and quality of performance (e.g., Brünken et al. 2002; Starcke et al. 2011).

A growing body of research indicates that media multitasking is negatively associated with cognitive performance and respective cognitive and academic outcomes (Jeong and Hwang 2016; May and Elder 2018), although evidence for the direction of causality is still lacking (van der Schuur et al. 2015). Studies on the cognitive profiles of heavier/high media multitaskers (HMMs) compared to lighter/low media multitaskers (LMMs) indicate that HMMs show reduced performance in different cognitive domains, especially in working memory and inhibitory control, however, results are mixed regarding other domains, such as task switching or interference control (Uncapher et al. 2017). For multitasking behavior in general, executive functions have a special relevance. For example, individuals with dysfunctions in the prefrontal cortex (associated with executive functioning) perform poorly when being confronted with multiple tasks (e.g., Dreher et al. 2008). In their well-established multifaceted (unity/diversity) model of executive functions, Miyake and colleagues (Miyake and Friedman 2012; Miyake et al. 2000) proposed three core functions: shifting, updating, and inhibition. Shifting is defined as “switching flexibly between tasks or mental sets” (Miyake and Friedman 2012; p. 9). Updating refers to “constant monitoring and rapid addition/deletion of working memory contents” (ibid.), and inhibition is described as “deliberate overriding of dominant or prepotent responses” (ibid.). Studies indicate that a common executive function factor, but in particular updating and inhibition, contribute to multitasking behavior (Bühner et al. 2006; Himi et al. 2019). Besides executive functions, other cognitive components including working memory capacity, attention control, and fluid intelligence were found to predict multitasking ability (Bühner et al. 2006; Redick et al. 2016). On the neural level, a common processing network (involving the intraparietal sulcus, right anterior insula, and left dorsal premotor cortex) was identified to be crucially involved in different types of multitasking (Worringer et al. 2019). The authors assume processes associated with the allocation of attention, action intentions, and effort for achieving the task goals to underlie multitasking. Accordingly, multitasking appears to be associated with a specific cognitive profile, which is why we assume differences in cognitive abilities between HMMs and LMMs. These profiles might also cause differences in domains that relate to similar underlying cognitive abilities, such as decision-making.

Cognitive abilities associated with multitasking, including working memory and different executive functions, were also shown to contribute to advantageous decision-making in risky situations (e.g., Schiebener and Brand 2015b). The current study, therefore, aimed to investigate differences between HMMs and LMMs regarding decision-making abilities and associated cognitive functions (executive functions, working memory, fluid intelligence, and attention-related deficits). Furthermore, we measured facets of impulsivity and self-control as well as participants’ expectancies toward media multitasking. In addition, multitasking with media nowadays probably involves the use of online applications. As excessive/problematic Internet use is associated with deficits in the investigated cognitive domains including executive functions, attention, and decision-making (Ioannidis et al. 2019) as well as with heightened impulsivity (Lee et al. 2012), we exploratively looked at media multitaskers’ tendencies toward problematic Internet use as an indicator of negative consequences resulting from excessive/dysfunctional media use.

### Decision-making abilities

Neurocognitive decision-making literature often distinguishes between two types of decision situations: decisions under ambiguity/ambiguous risk and decisions under objective risk (Brand et al. 2006; Schiebener and Brand 2015a; Yates and Stone 1992). In decisions under ambiguous risk, no explicit information about the available options is provided. Instead, decision makers have to learn from feedback, which of the chosen options are more or less advantageous (Bechara et al. 1994; Damasio 1994). In experimental studies, such decisions are often assessed with the Iowa Gambling Task (IGT; Bechara et al. 1994, 2000). In the IGT, participants have to choose between four similar looking decks of cards for which the potential consequences (gains or losses of fictitious money) are unknown. To perform well, participants have to learn from the given feedback that two decks are more advantageous (leading to positive outcomes in the long run) than the others and adjust their choice behavior accordingly (Bechara et al. 1997; Steingroever et al. 2013). In contrast, decision situations under objective risk offer explicit information about the potential consequences of available options from the beginning, meaning that the possible (positive and/or negative) outcomes and their probabilities are given or calculable. Thus, decision makers do not necessarily need to learn from previous trials, rather, they can develop and apply calculations, systematic comparisons, or strategies from the very beginning (Brand et al. 2006; Schiebener and Brand 2015a). One of the most frequently used tasks to measure decision-making performance under objective risk is the Game of Dice Task (GDT; Brand et al. 2005). In this computerized task, participants have to bet multiple times, on which number will occur on the next virtual die-roll. The bet can be placed on one of four classes of options. Two of them are of high risk (i.e., the chance to win is lower than the risk to lose) and thus disadvantageous in the long run, while the other two classes of options are of low risk (i.e., the chance to win is equal or higher than the risk to lose) and thus advantageous.

Performance in the IGT (when considering all 100 trials) was correlated, in some but not all studies, with tests of executive functions (Toplak et al. 2010). Several studies suggest that a substantial part of the variance in IGT performance seems to be related to emotional learning from feedback (Bechara et al. 1994, 1997; Denburg et al. 2001). Good performers in the task begin to produce gut feelings (or “intuitions”) that can guide decisions toward choosing advantageous or avoiding disadvantageous decks (Denburg et al. 2007). Such gut feelings may guide advantageous behavior even before explicit knowledge about the contingencies of the decision situation (Bechara et al. 1997; but see also Buelow and Suhr 2009; Dunn et al. 2006; Maia and McClelland 2004). However, in considering the performance of only the last 60 trials (in which the attributes of the cards have already been learned/understood) studies show consistent correlations with executive functions and reasoning abilities (Brand et al. 2007b; Kim et al. 2011; Schiebener and Brand 2016). These cognitive functions are not only involved in feedback processing, but also in reflecting about options, planning strategies as well as controlling impulses, all of which become more important after the rules of the task have been detected (Brand et al. 2007b; Schiebener and Brand 2016).

Performance in the GDT is consistently correlated with different tests of executive functions, reasoning, and working memory (Brand et al. 2009; Schiebener and Brand 2015a; Schiebener et al. 2011; Wood et al. 2016). Neurological patients with deficits in executive functions often make highly risky (i.e., very disadvantageous) decisions in the GDT (Brand et al. 2004; Delazer et al. 2007; Euteneuer et al. 2009). Also, persons with excessive media use (problematic online gaming) perform worse than controls on the GDT (Pawlikowski and Brand 2011). Several cognitive functions are relevant for successfully completing the task, including those involved in ratio processing, planning and applying rational decision-making strategies, and controlling the impulse to choose alternatives that offer high gains but are very risky (Brand et al. 2006; Schiebener and Brand 2015a).

In sum, the main difference between decisions under explicit risk (GDT) and decisions under ambiguous risk (IGT) is that, in decisions under explicit risk, contingencies are provided, while, in decisions under ambiguous risk, they need to be learned from feedback. Cognitive abilities including working memory and executive functions were shown to be relevant for making advantageous decisions in both types of decision situations.

### Cognitive functions and impulsivity in media multitaskers

Although results are mixed regarding some domains of cognitive functioning, empirical findings indicate decreased cognitive performance in HMMs compared to LMMs (Uncapher et al. 2017). HMMs, as compared to LMMs, are reported to show reduced working memory capacity (Cain et al. 2016; Sanbonmatsu et al. 2013; Uncapher et al. 2016), reduced fluid intelligence (Minear et al. 2013), difficulties in interference control and in filtering out distracting information (Cain and Mitroff 2011; Moisala et al. 2016; Sanbonmatsu et al. 2013), and difficulties in sustaining attention (Cain and Mitroff 2011). However, others did not find any relations between heavy media multitasking and deficits in working memory (Minear et al. 2013; Ophir et al. 2009) or sustained-attention (Ralph et al. 2015). Mixed findings also appear with respect to task-switching abilities, with some reporting a positive relation (Alzahabi and Becker 2013), while others report a negative (Ophir et al. 2009) or no relation (Minear et al. 2013) with heavy media multitasking. It should be noted that there is no clarity about the cause-and-effect relationship between media multitasking and cognitive functions (Carrier et al. 2015; Uncapher et al. 2017). Based on research on general multitasking behavior (e.g., Bühner et al. 2006; Himi et al. 2019; Redick et al. 2016), it can be assumed that executive functions (especially updating and inhibition), working memory, attention, and fluid intelligence may also contribute to media multitasking.

Regarding impulsivity, several studies demonstrated that HMMs report higher levels of impulsivity than LMMs (Minear et al. 2013; Sanbonmatsu et al. 2013; Yang and Zhu 2016). In the study by Ophir et al. (2009), higher attentional impulsivity predicted decreased working memory performance as well as heavier media multitasking. Recently, Shin et al. (2019) had a closer look at different dimensions of impulsivity in media multitaskers by use of experimental paradigms assessing impulsivity and inhibitory control. The results showed associations between media multitasking and high attentional impulsivity, but no associations with inhibitory control. The authors suggest that HMMs tend to inattention (slow and erroneous reactions in go/no-go task), but show superior inhibitory control of already initiated reactions (stop-signal task). According to Dickman (2000), individuals with high impulsivity are good at tasks that require quick shifting of attention, but they have difficulties in fixing attention to the relevant source of information. Moreover, high levels of impulsivity are associated with tendencies toward short-term oriented, risky, and disadvantageous decision-making in laboratory tasks (e.g., Bayard et al. 2011; Buelow and Suhr 2013; Mueller et al. 2017) as well as with tendencies toward problematic use of (online) media, such as Internet games (Bargeron and Hormes 2017), smartphones (Kim et al. 2016), or social media (Wu et al. 2013).

High impulsivity as well as deficits in executive functions (including updating and inhibition), attention, and decision-making are also associated with problematic Internet use (Ioannidis et al. 2019; Lee et al. 2012). Problematic Internet use is a phenomenon with potential clinical relevance (e.g., Kuss et al. 2014; Young 1998) as it is probably related to addictive behaviors such as gaming disorder (World Health Organization 2019). It denotes the uncontrolled and continued use of Internet applications despite the occurrence of negative consequences in private, social, and professional aspects of life (Brand et al. 2016). Besides the presumption that media multitasking often includes activities performed online, such as information search, streaming of music/films/television, learning, or social communication (including video-chats), different cognitive profiles of HMMs and LMMs may also reflect differences in predispositions toward a problematic use of online media. Thus, the current study additionally investigates tendencies toward problematic Internet use in HMMs compared to LMMs as an indicator of negative consequences resulting from high usage of (online) media.

### Media multitasking expectancies and problematic Internet use

Hwang et al. (2014) identified habits, the wish for information, social contact, enjoyment, and the hope to increase efficiency as major motives for multitasking with media. In other words, engaging in media multitasking seems to be linked with several positive expectancies. This fits well with the uses and gratifications approach, suggesting that media consumers use media to address their social and psychological needs (e.g., Katz et al. 1973; Zhang and Zhang 2012). In the context of excessive Internet use (independent of the medium), Brand et al. (2014a) showed that excessive users expect using the Internet to have positive effects on their emotional status. These positive expectancies include expectancies to experience positive feelings such as pleasure and fun, but also expectancies of the use helping to reduce negative feelings such as loneliness or to escape from problems (Brand et al. 2014a). Although media multitasking does not always involve the Internet, comparable expectancies can be assumed for multitasking with media. Beyond positive consequences, individuals may also expect potential negative consequences from the decision to multitask with media. To the best of our knowledge, there are no studies addressing expectancies of negative consequences of media multitasking. Therefore, in the current study, we aimed to measure not only positive but also negative media multitasking expectancies. To develop a respective scale, we adapted existing questionnaires such as the Internet-Use Expectancies Scale (Brand et al. 2014a) and formulated new items based on existing questionnaires assessing media use expectancies and motives (Hwang et al. 2014; Wang and Tchernev 2012).

### Research aims and hypotheses

The current study aims at adding to previous findings on whether cognitive functions HMMs differ from LMMs regarding performance in different cognitive function tasks and, moreover, in different types of decision-making tasks.

Based on previous findings, we expect HMMs, as compared to LMMs, to show reduced cognitive performance and higher levels of trait impulsivity. Moreover, given the relevance of cognitive functions for advantageous decision-making under different risk conditions, we hypothesized that HMMs perform worse than LMMs in both the GDT and the IGT (particularly in the last 60 trials). Regarding expectancies, we assumed HMMs to expect more positive and less negative consequences from media multitasking than LMMs do. Lastly, we assumed that HMMs display a higher tendency toward problematic Internet use than LMMs.

## Method

### Participants

All participants participated voluntarily and ethical guidelines of the declaration of Helsinki were fulfilled. The study was approved by a local ethics committee. Informed consent was obtained from all individual participants included in the study. All participants were recruited via local advertisement and completed an online pre-study. Thereafter, those who were identified as LMMs and HMMs were invited to our laboratory to conduct experimental tasks on decision-making and executive functions as well as additional questionnaires.

### Online pre-study: determining LMMs and HMMs

In the online pre-study, an incidental sample of 182 participants (141 females), aged 17–52 years (*M* = 21.04, *SD* = 4.56 years), completed the Media Multitasking Index (MMI) in a slightly adapted version of Ophir et al. (2009). The present version of the MMI addresses media multitasking in ten different forms (print media, short-message services, text-based websites, social networks, music, telephone-/video-chat, television, video games, homework/learning/work, face-to-face conversation). For each medium, there were eleven questions: (1) How many hours of an average day is the activity carried out? (2–11) What percentage of the time is each of the ten activities carried out in parallel to the activity asked for in (1)?. Besides question (1), where a free number had to be entered, participants used sliders for providing their answers. In contrast to the original version of the MMI, we used 10 instead of 12 media activities and used a slider allowing the participants to directly enter their percentages instead of using a four-point scale that is then converted into percentages (Ophir et al. 2009). Given these adaptations, the final MMI score is not comparable to those reported in previous studies. However, the identification of LMMs and HMMs remains the same: For each participant, an overall score was calculated using the formula by Ophir et al. (2009). The MMI score ranged from 0.22 to 5.04. The mean MMI score of 1.79 (SD = 0.91) was lower compared to previous studies (e.g., Lui and Wong 2012; Ophir et al. 2009) but showed a similar right-skewed distribution. By forming groups of 1SD above/below the mean, we determined 22 LMMs (*M* = 0.60 ± 0.16) and 31 HMMs (*M* = 3.31 ± 0.56) and invited only those to a follow-up study in the laboratory.

### Sample of the laboratory study

Out of the invited individuals, *n* = 44 (36 females; aged 18–52 years, *M* = 22.44, SD = 8.04) participated in the laboratory study. The sample consisted of 19 LMMs (14 females, mean age: *M* = 26.37, SD = 11.25) and 25 HMMs (22 females, mean age: *M* = 19.52, SD = 1.56). The two groups did not differ regarding gender distribution (χ^2^(1, N = 44) = 1.49, *p* = 0.223) but they differed regarding age, *t*(18.53) = 2.64, *p* = 0.017, *d* = 0.85. Consequently, we integrated age as a covariate in all of the statistical analyses. Additionally, as to be expected, the two groups significantly differed regarding the MMI (LMMs: *M* = 0.68 ± 0.32; HMMs: *M* = 3.24 ± 0.53), *t*(39.89) = − 19.80, *p* < 0.001, *d* = 5.83. All participants were investigated in a 1:1 laboratory setting.

## Instruments

### Decision-making abilities

The IGT was used as a measure of decision-making under ambiguous risk. In the computerized IGT (Bechara et al. 1994, 2000), participants are asked to select one of four decks of cards (A′, B′, C′, or D′) per trial. The decks contain cards that imply the win/loss of virtual money. Over a total of 100 trials, participants try to maximize their outcome, but without being told that two decks (C’ and D’) are more advantageous than the others (A’ and B’). Following the convention, we used the net scores ([C′ + D′]  −  [A′ + B′]) as measures of IGT performance. Higher net scores indicate higher preference for the advantageous decks indicating better decision-making performance. For the following analyses, we used the overall net score (over all 100 trials) as well as net scores separated for the first 40 and the last 60 trials (Brand et al. 2007b).

The GDT (Brand et al. 2005) was used to measure decision-making under objective risk. The GDT is a computerized dice task. Participants are instructed to maximize a virtual capital of €1,000 by betting on the results of consecutive die rolls. In each of the 18 rounds, the participant can choose to bet on a single number or on combinations of two, three, or four numbers. Bets on few numbers (implying a high risk) is associated with high gain/loss amounts, while bets on combinations of numbers are associated with smaller possible gain/loss amounts (but present lower risk). For each option, possible gains/ loss amounts are presented explicitly on the screen. They have the following contingencies: (1) one number (e.g., “1” or “6,” gain/loss = €1,000; winning probability = 0.167), (2) two numbers (e.g., “3, 4,” gain/loss = €500; winning probability = 0.333), (3) three numbers (e.g., “1, 2, 3,” possible gain/loss = €200; winning probability = 0.500), (4) four numbers (e.g., “3, 4, 5, 6,” possible gain/loss = €100; winning probability = 0.667). Betting on one or two numbers is classified as very risky/disadvantageous, because the winning probability is lower than the loosing probability. Betting on three and four numbers is classified as less risky/advantageous, because the winning probability is at least as high as the loosing probability. As a measure of GDT performance, the net score is used (i.e., n[advantageous] – n[disadvantageous]) which represents decision-making performance. Additionally, the number of choices for one single number indicating high-risk choices is used.

### Fluid intelligence

Logical reasoning was assessed by use of Subtest 4 of the “Leistungsprüfsystem” (LPS4), a German intelligence test battery by Horn (1983). The LPS4 comprises 40 rows each representing a logical sequence of 9 numbers and/or letters, in which one letter/number contradicts the logical order. Participants are asked to indicate the mismatching character in as many rows as possible within a time frame of 8 min. The sum of correct rows is used as outcome parameter.

### Executive functions

We used three different measures of executive functions to account for the diversity of executive functions (Miyake et al. 2000). The used measures share common variance (unity) and cannot be assigned to one function exclusively, however, each task places emphasis on either shifting, updating, or inhibition (diversity).

General executive functions, were measured using the Modified Card Sorting Test (MCST) which depicts a modified computerized version of the card sorting test by Nelson (1976), which especially loads on shifting (Miyake et al. 2000). In the MCST, the number of errors is counted separately for perseverative and non-perseverative errors and is used as an inverse measure of good executive functioning.

The Trail Making Test (TMT, parts A and B) by Reitan (1958) was used to assess updating (/cognitive flexibility) and information processing speed. In this paper–pencil task, participants have to join letters (part A) as well as letters and numbers alternatingly (part B) with a drawn line. The seconds needed serve as an inverse performance measure.

Additionally, we used the Stroop test, also known as Color-Word Interference Test (Stroop 1935), mainly representing inhibition. As in the TMT, high scores (i.e., seconds needed to complete the task) represent lower performance in the respective domain.

### Working memory capacity

Working memory span was assessed using the DigitSpan backwards test (Kessler et al. 2000), which requires the backwards repetition of verbally presented sequences of digits. The test starts with sequences of two digits, which increase by one as soon as one out of two trials of the similar number of digits correct. The length of the correctly expressed sequence serves as a measure of working memory capacity.

### Trait impulsivity and self-control

Impulsivity was assessed with the German 15-item version of the Barratt Impulsiveness Scale 11 (Meule et al. 2011; Patton et al. 1995). The self-report scale has three subscales: motor impulsivity (e.g., “I do things without thinking.”), attentional impulsivity (e.g., “I don’t pay attention.”), and non-planning impulsivity (e.g., “I plan tasks carefully.” inverted item). The items have to be answered on a 4-point Likert scale ranging from 1: “rarely/never” to 4: “almost always/always.”

Self-control was assessed with the German short version of the Self-Control Scale (Bertrams and Dickhäuser 2009; Tangney et al. 2004). The scale has 13 items (e.g., “I’m good at resisting temptations.”) to be answered on a five-point Likert scale (from 1: “not at all” to 5: “very much”). The scale has a single factor structure.

### Dysfunctional behavioral consequences

Additionally, we used the Attention-Related Cognitive Errors Scale (ARCES; Cheyne et al. 2006). The ARCES is a self-report measure assessing dysfunctions in everyday performance due to attentional deficits. The scale consists of twelve items, each answered on a five-point Likert scale.

We used the short Internet-Addiction-Test (s-IAT; Pawlikowski et al. 2013) to assess subjective impairments in everyday functioning due to an uncontrolled/addictive use of online applications, also referred to as problematic Internet use. The s-IAT consists of 12 items to be answered on a five-point Likert scale (from 1: “never” to 5: “very often”). It has two subscales: “Loss of control/time management” due to Internet use (e.g., “How often do you find that you stay online longer than you intended?”) and “craving/social problems” (e.g., “How often do you feel depressed, moody, or nervous when you are offline, which goes away once you are back online?”). All tests and questionnaires were analyzed according to the respective typical conventions.

### Media multitasking expectancies

Expectancies about media multitasking were measured by the Media Multitasking Expectancies Scale, developed with respect to different a priori defined types of consequences derived from the literature regarding media multitasking motives (Hwang et al. 2014), Internet-use expectancies (Brand et al. 2014a) as well as from own considerations of the authors. We addressed positive expectancies (e.g., immediate reinforcement, fun experience, increase of efficiency) and negative expectations (e.g., cognitive impairments/ distractions, stress, social sanctions) for adding a medium to an ongoing activity. Both the positive and negative expectancies subscales consist of four further sub-facets each. “Positive reinforcement” (4 items) and “avoidance expectancies” (4 items) are two facets of positive expectancies which are derived from the Internet-Use Expectancy Scale (Brand et al. 2014a) and which were adapted for media multitasking. Exemplary items are as follows: “I use media in parallel with other activities or media because it enables/facilitates to experience pleasure.” (positive reinforcement) and “I use media in parallel with other activities or media because it enables/facilitates to distract from problems.” (avoidance expectancies). Additionally, two newly developed subscales were used: “pastime to reduce boredom” (5 items, e.g., “I use media in parallel with other activities or media because it enables/facilitates to kill time.”, Cronbach’s Alpha = 0.95) and “efficiency” (5 items, e.g., “I use media in parallel with other activities or media because it enables/facilitates to get things done quicker.”, Cronbach’s Alpha = 0.95). Negative expectancies were measured with items referring the following sub-facets: “cognitive impairments” (9 items, e.g., “When I use media in parallel with other activities or other media, I miss much of one of the activities/media.”, Cronbach’s Alpha = 0.93), “stress/effort” (6 items, e.g., “When I use media in parallel with other activities or other media, I get stressed out.”, Cronbach’s Alpha = 0.94), and “social sanctions” (6 items, e.g., “When I use media in parallel with other activities or other media, other people get annoyed.”, Cronbach’s Alpha = 0.90). All items of the Media Multitasking Expectancies Scale (MMES) had to be answered on a six-point Likert scale ranging from 1: “completely disagree” to 6: “completely agree.” Thereafter, we asked which medium participants had primarily in mind while answering the questions.

Moreover, the general urge/desire to multitask with media was measured with four newly developed items which had to be answered on a six-point Likert scale (1: “totally disagree” to 6: “completely agree”). The four items asked, in different wordings, how often and strong respondents desire to add a(nother) medium to an ongoing activity (e.g., “During an activity or during usage of a medium, I often feel a strong urge/desire to turn toward a(nother) medium”). The subscale had a high internal consistency (Cronbach’s Alpha = 0.93). Thereafter, we again asked which medium the participants had primarily in mind while answering the questions.

English translations of all items can be found in the “Appendix” together with the items of all other newly developed scales (which are described below).

## Results

First, we compared HMMs and LMMs regarding facets of impulsivity and self-control. Secondly, we tested the hypothesized performance differences in the decision-making tasks (IGT and GDT) and cognitive function tests (LPS4, MCST, TMT, Stroop, DigitSpan) as well as regarding self-report measures of attention-related cognitive errors (ARCES), problematic Internet use (s-IAT), and media multitasking urge and expectancies. Differences were calculated using ANCOVAs including group (HMMs/LMMs) as between-subjects factor and age as covariate in each of the analyses.

### Differences in decision-making and cognitive functions

The results of the comparison of HMMs and LMMs concerning the decision-making performance in the IGT and GDT, respectively, are shown in Table 1. HMMs made significantly less advantageous decisions in the IGT than LMMs as indicated by a lower overall net score and a lower net score of the last 60 IGT trials. No differences were observed regarding the first 40 IGT trials. HMMs and LMMs did not differ in GDT performance.

**Table 1:** Comparison of HMMs and LMMs regarding decision-making performance

	LMMs (*n* = 19)	HMMs (*n* = 25)	ANCOVA		
	*M* (SD)	*M* (SD)	*F(1,41)*	*p*	*η*^2^_*p*_
IGT: overall	19.79 (25.00)	0.72 (18.12)	6.63	.014	.139
IGT: first 40 trials	2.00 (10.87)	− 2.32 (5.44)	2.01	.164	.047
IGT: last 60 trials	17.79 (18.61)	3.04 (14.52)	6.98	.012	.145
GDT: net score	7.47 (11.90)	9.88 (8.44)	0.56	.459	.013
GDT: one single number	1.47 (3.31)	1.80 (3.75)	0.03	.861	.001

*LMMs* Low media multitaskers, *HMMs* High media multitaskers, *IGT* Iowa Gambling Task, *GDT* Game of Dice Task. Covariate age did not have any significant effects

Table 2 shows the results of the group comparisons regarding performance measures of cognitive functions. HMMs as compared to LMMs performed significantly weaker in tests assessing fluid intelligence (LPS4) and working memory capacity (Digit span backwards). Both effects were of medium sizes. Regarding executive functions, none of the differences were significant. On a descriptive level, HMMs on average performed weaker in the MCST than LMMs. Contrarily, HMMs tended to superior (i.e., faster) performance in the TMT and Stroop. But, again, the effects were not significant and of small sizes only (see Table 2). As might be expected, the covariate age had significant effects on all of the reported significant differences.

**Table 2:** Comparison of HMMs and LMMs regarding measures of cognitive functions

	LMMs (*n* = 19)	HMMs (*n* = 25)	ANCOVA		
	*M* (SD)	*M* (SD)	*F(1,41)*	*p*	*η*^2^_p_
*Fluid intelligence*					
LPS4: correct answers	30.84 (4.00)	30.08 (3.00)	5.86	.020	.125
*Executive functions*					
MCST: non-perseverative errors	5.95 (5.92)	6.04 (5.82)	1.21	.279	.029
MCST: perseverative errors	0.89 (1.63)	0.96 (1.97)	1.36	.250	.032
TMT A: time (in sec.)	24.27 (10.28)	22.28 (7.70)	2.55	.118	.059
TMT B: time (in sec.)	52.22 (22.44)	47.30 (15.28)	1.43	.239	.034
TMT Difference: B-A	27.95 (15.79)	25.02 (13.62)	0.26	.610	.006
Stroop: time (in sec.)	65.40 (13.86)	58.58 (10.60)	0.38	.543	.009
Stroop: errors	0.42 (0.77)	0.60 (1.08)	0.15	.699	.004
*Working memory capacity*					
Digit span backwards	5.32 (0.89)	5.12 (0.78)	4.46	.041	.098

LMMs, Low media multitaskers; HMMs, High media multitaskers; LPS4, subtest 4 (logical reasoning) of the Leistungsprüfsystem; MCST, Modified Card Sorting Test; TMT, Trail Making Test. Covariate age had significant effects on all measures except Stroop errors

### Differences in impulsivity and self-control

Regarding facets of impulsivity, HMMs on average showed higher impulsivity than LMMs, especially regarding motor and attentional impulsivity, however, the differences were not statistically significant. Mean levels of self-control were slightly lower in HMMs compared to LMMs but also not significantly different (see Table 3).

**Table 3:** Comparison of HMMs and LMMs regarding impulsivity and self-control

	LMMs (*n* = 19)	HMMs (*n* = 25)	ANCOVA		
	*M* (SD)	*M* (SD)	*F(1,41)*	*p*	*η*^2^_*p*_
BIS non-planning	2.03 (0.54)	2.08 (0.71)	1.28	.265	.030
BIS motor	1.95 (0.43)	2.35 (0.69)	3.02	.090	.069
BIS attentional	2.03 (0.34)	2.31 (0.53)	3.55	.067	.080
Short Self-Control Scale	3.36 (0.44)	3.08 (0.60)	3.10	.086	.070

*LMMs* Low media multitaskers, *HMMs* High media multitaskers. *BIS* Barratt Impulsiveness Scale, Covariate age had a significant effect on BIS non-planning

### Differences in dysfunctional outcomes and media multitasking expectancies

Attention-related dysfunctions in daily life, as indicated by ARCES scores, were higher in HMMs (*M* = 2.47, SD = 0.71) compared to LMMs (*M* = 2.05, SD = 0.56), however, the difference was not significant, *F*(1,41) = 3.32, *p* = 0.076, *η*^2^_*p*_ = 0.075. The comparison regarding problematic Internet use shows a significant effect of large size, *F*(1,41) = 13.45, *p* = 0.001, *η*^2^_*p*_ = 0.247. HMMs (*M* = 28.52, SD = 8.42), as compared to LMMs (*M* = 18.74, SD = 5.88), had significantly higher s-IAT scores indicating higher functional impairments due to problematic use of online applications. In both analyses, the covariate age had no effect, *p*’s > 0.05.

Regarding the media multitasking expectancies, only two facets were significantly different between the groups. Most notably, HMMs, compared to LMMs, had more positive expectancies regarding negative reinforcement, meaning that they expected media multitasking more to help avoid negative feelings (see Table 4). Regarding the negative expectancies, HMMs compared to LMMs expected media multitasking to cause more social sanctions. All other media multitasking expectancies did not differ between groups (see Table 4). Most of the participants (77.3%) indicted to have had primarily the smartphone in mind while answering the expectancies questions (other percentages were as follows: 6.8% TV, 2.3% Notebook, 6.8% personal computer, 4.5% radio/music, 2.3% tablet computer).

**Table 4:** Comparison of HMMs and LMMs in expectancies regarding media multitasking

	LMMs	HMMs	ANCOVA		
Media multitasking expectancies	*M* (SD)	*M* (SD)	*F(1,41)*	*p*	*η*^2^_*p*_
Positive: positive reinforcement	2.88 (1.13)	3.63 (1.03)	2.53	.120	.058
Positive: avoidance expectancies	1.80 (0.78)	2.98 (1.13)	12.51	.001	.234
Positive: reduce boredom	4.27 (1.12)	5.14 (0.89)	3.90	.055	.087
Positive: increase efficiency	3.15 (1.35)	3.80 (1.15)	1.98	.167	.046
Negative: cognitive impairments	3.89 (0.97)	4.02 (0.86)	1.84	.183	.043
Negative: stress/effort	2.84 (1.07)	2.48 (1.27)	0.12	.732	.003
Negative: social sanctions	3.17 (1.07)	3.55 (0.89)	5.60	.023	.120

*LMMs* Low media multitaskers, *HMMs* High media multitaskers. Covariate age had significant effects on negative expectancies: cognitive impairments (*p* = .036) and social sanctions (*p* = .013)

Regarding the urge/desire to multitask with media, HMMs (*M* = 3.46, SD = 1.17) scored higher than LMMs (*M* = 2.74, SD = 1.21), but the difference was not significant, *F*(1,41) = 2.39, *p* = 0.130, *η*^2^_*p*_ = 0.055. The covariate age had no effect. When answering the urge/desire questions, again, the majority of participants (65.9%) indicted to have had primarily the smartphone in mind (other percentages were as follows: 9.1% TV, 6.8% Notebook, 4.5% personal computer, 4.5% radio/music, 2.3% tablet computer).

## Discussion

The current study investigated differences in risky decision-making (assessed using the IGT and GDT), cognitive functions (fluid intelligence, executive functions, working memory), impulsivity, and dysfunctional behavioral consequences between HMMs and LMMs. Based on previous findings which indicate that HMMs have deficits in cognitive functions that are also relevant for making advantageous decisions (e.g., executive functions and working memory), we expected HMMs to perform weaker in both decisions under ambiguous risk (IGT) and decisions under objective risk (GDT). The results show that HMMs performed weaker than LMMs in the IGT but not in the GDT. Although the sample size was relatively small, the results point at potential deficits in decision-making under ambiguous risk but not under objective risk. The IGT requires participants to learn the consequences and risks of their behavior from feedback (e.g., Bechara et al. 1997). In contrast, the GDT provides participants with information that allow to judge probabilities and to apply strategies from the very beginning of the task, without the necessity to process feedback (e.g., Brand et al. 2005). The results suggest that HMMs might have problems with learning from the consequences of their behavior.

Based on previous studies, we expected that HMMs perform weaker than LMMs in tests of working memory, general executive functions, and reasoning. The results supported our hypothesis with regard to working memory and fluid intelligence, which is in line with previous findings (Cain et al. 2016; Minear et al. 2013; Sanbonmatsu et al. 2013; Uncapher et al. 2016). The differences were of medium effect sizes. Differences in executive functions were not confirmed in the current sample. Looking at effect sizes, one might speculate that differences, if any, in TMT performance became apparent if the sample size would have been larger. However, executive functions do not appear to play an influential role, which is also indicated by the fact that the decision-making performance under objective risk (GDT) did not differ between groups, although it is closely associated with performance in the executive functioning tests used in the current study (Brand et al. 2009; Schiebener et al. 2014). In many studies with psychiatric and neurological patients, impairments in the GDT occurred particularly in case of impairments in executive functions, but not in case of intact executive functions (Brand et al. 2007a; Delazer et al. 2007; Euteneuer et al. 2009). The current study investigated a non-clinical sample, in which HMMs overall showed average or above average performance in the applied cognitive tasks. According to others (e.g., van der Schuur et al. 2015) HMMs are assumed not to have any general deficits in executive control but maybe more specific ones. For example, it was suggested that HMMs have difficulties with blocking out irrelevant information (Cain and Mitroff 2011; Sanbonmatsu et al. 2013), while they have no difficulties or potentially even advantages in task switching or response inhibition (Alzahabi and Becker 2013; Minear et al. 2013). The results of the current study do not directly support this view, however, on a descriptive level the results indicate advantages of HMMs compared to LMMs in executive functions associated with information processing and inhibition/interference control (TMT and Stroop) but opposite tendencies regarding higher-order functions (as measured by the MCST, e.g., categorization and feedback-processing) and logical reasoning. Overall, the results add to the inconsistent findings regarding cognitive functions in extensive media multitaskers.

Based on literature from general multitasking behavior, superior performance in updating and inhibition skills are associated with higher multitasking, while shifting appeared to be less important (Bühner et al. 2006; Himi et al. 2019). On a descriptive level, the current results are in line with this view. However, we found that fluid intelligence and working memory capacity were significantly lower in HMMs, although these functions have been identified as important predictors for multitasking ability (Bühner et al. 2006; Redick et al. 2016). Accordingly, HMMs seem to show a different cognitive profile than high (general/non-media) multitaskers.

In contrast to other studies (e.g., Minear et al. 2013) we found no differences between HMMs and LMMs regarding measures of impulsivity and self-control. The mean scores were slightly higher for HMMs, especially with respect to attentional impulsivity. However, differences were not significant. Because of the heterogeneous groups of HMMs and LMMs, we controlled for age in any of the analyses, which may have reduced the effect of impulsivity. Although not significant and highly speculative because of the small sample size, it might be assumed that HMMs tend to higher attentional impulsivity and tend to good performance in tasks that require rapid attentional shifting (e.g., TMT and Stroop), but they performed weaker in tasks that require focused attention (e.g., logical reasoning test). This would be in line with the attentional-fixity theory (Dickman 1993, 2000) assuming that high impulsivity, in contrast to low impulsivity, is associated with the ability to easily shift attention from its current fixation.

The reported expectancies toward multitasking with media differed between HMMs and LMMs in the expected directions. HMMs expect more positive consequences from media multitasking, in particular that media multitasking leads to the reduction of unpleasant feelings. Positive expectancies are assumed to lead to a higher urge and (spontaneous) approach behavior (Breiner et al. 1999). However, HMMs did not show a significantly higher urge toward media multitasking. Interestingly, HMMs reported also more negative expectancies than LMMs but only regarding expected social sanctions of media multitasking behavior. On average, both groups judged the negative consequences to be moderate (so neither very high nor very low). Thus, the difference in expectations between LMMs and HMMs is mainly due to HMMs expecting more gratification (unburdening from negative feelings) from media multitasking, which fits well with uses and gratifications approaches to media consumption (Katz et al. 1973).

Furthermore, HMMs tend to impairments in everyday functioning as indicated by a trend toward more attention-related dysfunctions and a significantly higher tendency toward problematic Internet use, as compared to LMMs. A recent meta-analysis revealed that problematic Internet use is associated with deficits in cognitive functions including working memory and decision-making (Ioannidis et al. 2019). HMMs appear to have deficits in similar domains. Furthermore, HMMs tend to higher impulsivity (Minear et al. 2013), which was also reported for individuals with problematic Internet use (Lee et al. 2012). This is also in accordance with theoretical models on problematic Internet-use behaviors, such as the “Interaction of Person, Affect, Cognition, Execution” (I-PACE) model (Brand et al. 2019, 2016). Furthermore, the I-PACE model suggests that, in early stages, the repeated reward experience due to the use of specific applications forms reward expectancies and specific coping styles (e.g., perform the behavior to reduce stress or negative feelings) which in turn increase the likelihood to perform the behavior again and again. In later stages, the use becomes more habitual and is associated with affective and cognitive biases as well as craving reactions making it increasingly difficult to control the behavior (Brand et al. 2019). Consistently, we found high (compared to low) media multitaskers to show higher reward expectancies and a tendency toward higher urges to perform the behavior. It should be noted that the current study is incapable of clarifying the direction of the relationship between media multitasking and problematic Internet use. Accordingly, problematic Internet use might increase the tendency to multitask with media or vice versa. Also, several other relationships are conceivable (reciprocal effects, third-party variables affecting symptoms of pathological Internet use and media multitasking, etc.). Nevertheless, it might be assumed that the differences in cognitive functions between HMMs and LMMs may partly be explained by the difference in problematic Internet use. Unfortunately, this study’s sample was too small to analyze differences in cognitive profiles between HMMs with and without problematic Internet use. We would hypothesize that HMMs without problematic use behavior show superior cognitive functions (reflecting advantages in multitasking) compared to those with problematic use behavior (associated with deficits in cognitive functions). We recommend future studies to further investigate these relationships.

In summary, the current study gives first hints for excessive media multitasking being associated with reduced decision-making performance in case the decisions require learning from feedback. Excessive media multitasking was also associated with strong desires to multitask with media and higher positive expectancies from media multitasking. HMMs showed slight decreases in fluid intelligence and working memory capacity, but, advantages in information processing might be assumed. No differences were found regarding trait impulsivity and self-control, at most there was a small trend toward higher attentional impulsivity in HMMs. Finally, high media multitasking was related to more problematic Internet use.

Some limitation should be mentioned when interpreting the results of the current study. A major limitation is the small sample size in the laboratory study. For this reason, the results should be treated with particular caution. Nevertheless, the results provide valuable insights and first hints for further future investigations. Another limitation is the heterogeneity of the two groups with respect to sociodemographic variables. Although we controlled for age differences, future studies may implement a better matching of to-be-compared groups. Due to the small sample size and the included covariate, the results should be especially treated with caution. Finally, we like to again note that the current study does not allow any conclusions regarding cause-and-effect relationships between media multitasking and the other measures (decision-making, cognitive functions). To clarify the aspect of causality, future studies need to use methods other than comparing HMMs and LMMs ex-post-facto.

## Appendix

### A1: Questionnaire used to assess urge/desire to multitask with media

The following questions relate to situations in which you are carrying out an activity (e.g., a face-to-face conversation, driving a car, learning, working, doing homework, etc.) or in which you are using a medium (e.g., computer, TV, etc.).

**Table:**

	Completely disagree − − −	− −	−	+	+ +	Completely agree + + +
During an activity or during usage of a medium I often feel a strong urge/desire to turn toward a(nother) medium						
The urge to turn toward a(nother) medium becomes very intense						
Turning toward a(nother) medium happens as if automatized						
My urge toward multitasking with media is very intense						

When answering these questions, which medium did you have in mind primarily?: _________.

### A2: Questionnaire used to assess positive media multitasking expectancies.

I use media in parallel with other activities or media, because it allows/facilitates…

**Table:**

		Completely disagree − − −	− −	−	+	+ +	Completely agree + + +
Positive reinforcement	…to experience pleasure						
	…to have fun						
	…to gain positive emotions						
	…to feel good						
avoidance expectancies	…to distract from problems						
	…to escape from reality						
	…to avoid loneliness						
	…to avoid annoying duties						
Pastime to relief boredom	…avoid boredome						
	…while away the time						
	…bridge time						
	…bear waiting periods						
	…have something to do while I’m waiting						
Efficiency	…get done more in the same time						
	…complete more tasks						
	…take care for all my tasks						
	…save time						
	…get things done more quickly						

### A3: Questionnaire used to assess negative media multitasking expectancies.

When I use media in parallel with other activities or media …

**Table:**

		Completely disagree − − −	− −	−	+	+ +	Completely agree + + +
Cognitive impairments	…I can hardly concentrate on all activities/media						
	…I miss a lot of one of the activities/media						
	…my attentional performance drops						
	…it harms my concentration						
	…I easily forget the other activity/medium						
	…it strongly distracts from one of the activities/media						
	…I lose the concentration for one of the activities/media						
	…I can concentrate on only one of the activities/media anyway						
	…I fail to notice important things						
Stress	…it is exhausting						
	…it can easily overstrain me						
	…it stresses me out						
	…it exhausts me						
	…I feel depleted afterwards						
	…it quickly annoys me						
	…I’m more stressed afterwards than I was before						
	…I experience it as a burden						
Social sanctions	…it annoys other persons						
	…it is experienced as impolite						
	…other people become angry						
	…others are upset						
	…others receive a negative impression of me as a person						
	…others get annoyed by me						

When answering these questions, which medium did you have in mind primarily?: _________.
